# The Technical Efficiency of Specialised Milk Farms: A Regional View

**DOI:** 10.1155/2014/985149

**Published:** 2014-06-22

**Authors:** Jindřich Špička, Luboš Smutka

**Affiliations:** ^1^Faculty of Business Administration, Department of Business Economics, University of Economics, Prague, W. Churchill Square 4, 130 67 Prague 3, Czech Republic; ^2^Faculty of Economics and Management, Department of Economics, Czech University of Life Sciences Prague, Kamýcká 129, 165 21 Prague 6, Czech Republic

## Abstract

The aim of the article is to evaluate production efficiency and its determinants of specialised dairy farming among the EU regions. In the most of European regions, there is a relatively high significance of small specialised farms including dairy farms. The DEAVRS method (data envelopment analysis with variable returns to scale) reveals efficient and inefficient regions including the scale efficiency. In the next step, the two-sample *t*-test determines differences of economic and structural indicators between efficient and inefficient regions. The research reveals that substitution of labour by capital/contract work explains the variability of the farm net value added per AWU (annual work unit) income indicator by more than 30%. The significant economic determinants of production efficiency in specialised dairy farming are farm size, herd size, crop output per hectare, productivity of energy, and capital (at *α* = 0.01). Specialised dairy farms in efficient regions have significantly higher farm net value added per AWU than inefficient regions. Agricultural enterprises in inefficient regions have a more extensive structure and produce more noncommodity output (public goods). Specialised dairy farms in efficient regions have a slightly higher milk yield, specific livestock costs of feed, bedding, and veterinary services per livestock unit.

## 1. Introduction

Specialised dairy farms represent an important type of farming, but their importance varies within the EU. The share of milk production in specialised dairy farms within the total milk production in the EU ranges from 24% (Czech Republic) to 99.9% (some regions in Spain and Portugal). The rest of the milk production comes from mixed crop and livestock farms. Specialised farms are highly technologically demanding. They have to reach better yields and quality because they are unable to spread the price risk into various crops or commodities. Production efficiency is one of the key prerequisites for the competitiveness of enterprises in every business. The question about production efficiency of specialised dairy farms arises due to the expected abolition of the milk quota system in 2015.

The goal of this paper is to evaluate the production efficiency of specialised dairy farms among the FADN EU (farm accountancy data network EU) regions and to determine which structural and economic factors significantly affect the farming performance. Specialized dairy farms have not been a very important feature of Czech agriculture, since about three-quarters of total milk production is produced in large mixed crop and livestock agricultural enterprises. The importance of specialised dairy farms is significantly higher in Western Europe because they are often relatively small family farms with specialised agricultural production. The identification of production efficiency and its main determinants can reveal the weaker regions in the EU and show ways to improve their farming performance in the new Common Agricultural Policy after 2013.

This paper is organized as follows. After a review of the relevant literature about production efficiency in agriculture, the material and methods are described. The results describe and discuss the most important findings about the determinants of production efficiency of specialised dairy farms among EU regions. The conclusions indicate the purpose and the main findings.

This paper is based on experiences related to Czech agricultural production efficiency analyses conducted by a number of previous authors: Juřica et al. [1], Jelínek [2], Medonos [3], Davidova and Latruffe [4], Boudný et al. [5], Čechura [6, 7], and Malá [8]. A key element for the construction of this paper is derived from research conducted by Čechura [7]. Čechura identifies the key factors determining the efficiency of input use and the development of total factor productivity (TFP). He concludes that the developments in the individual branches are characterized by idiosyncratic factors, as well as the systemic effect, especially in animal production. The most important factors which determine both the technical efficiency and TFP are those connected with institutional and economic changes.

Machek and Špička [9] also apply the TFP approach in agriculture. They estimate total factor productivity of the agricultural sector based on firm-level accounting data. The results of the analysis suggest that the agricultural TFP growth does not necessarily move in the same direction as the growth of the economy.

It is also necessary to emphasize that agricultural enterprises, especially those in developed countries, are seriously affected by agricultural policy measures [10] or [11]. Technical efficiency analyses conducted by Bakucs et al. [10] proved that the applied subsidies have a direct (negative) impact on the efficiency of farms. Murova and Chidmi [11] evaluated the technical efficiency of farms under government programs in the USA, and the result of their research focused on the efficiency analysis of the federal milk marketing program which proved to have a significant and negative impact on technical efficiency.

Błazejczyk-Majka et al. [12] conducted some particularly interesting research on the technical efficiency analyses of EU farms, in which they used FADN data to find whether a higher specialisation and a bigger economic size class of farms determine a higher technical efficiency at the same scale for the farms from the new and old countries of the EU. The results recorded for mixed farms in relation to the pure technical efficiency indicate a greater efficiency for the farms from the “old” EU regions (EU-15) in comparison to the farms from the “new” regions, except for the largest farms. In relation to the new EU members, the results coming from the research conducted by Latruffe et al. [13] are important. Latruffe et al. analyse technical efficiency and its determinants for a panel of specialised crop and livestock production. The authors compare data envelopment analysis (DEA) with stochastic frontier analysis (SFA). They found that livestock farms are more technically efficient than crop farms and large farms are more efficient than small farms. The key determinants of efficiency are a degree of downstream market integration and soil quality.

Another very important driver of production efficiency—especially in the milk industry—is the technical efficiency [14]. Improvement in technical efficiency in milk production requires adequate and quality veterinary services, augmentation of feed and fodder resources at the farm, integration with a formal marketing system, and scaling-up of the dairy enterprise. Bardhan and Sharma [15] and Sajjad et al. [16] assess the technical efficiency of dairy farms using the stochastic frontier approach. They found that herd size, dry fodder, green fodder, concentrate/oil seed cake, hired labour, permanent labour, medicine and vaccination cost, and intensity of market participation are the major determinants that affected milk production. The increasing average age of livestock farmers and farmers' lack of experience also cause a decline in the efficiency.

Moreover, Binici et al. [17] revealed that if a farmer with average efficiency improved efficiency to that of the most efficient farmer in the sample, then the average dairy farmer could achieve significant cost savings. Moreover, age and contact with an extension officer have a positive impact on dairy production efficiency. Alemdar et al. [18] concluded that in the short run efficiency could be improved through methods (such as training) without requiring higher costs. However, in the long term structural enhancements such as the introduction of highbred milking animals would be required.

Nutrition and welfare determinants of production efficiency in dairy farming are also the object of many analyses: Uzal [19], Ryan et al. [20], Gourley et al. [21], Holtshausen et al. [22], Xue et al. [23], and Auldist et al. [24]. These authors all highlight the need for nutritional improvement of the cows' feed to improve the production and energy efficiency of milk production. Krause and Tondlova [25] emphasize the significance of environmental factors for the farms' competitiveness.

This paper is focused on structural and economic determinants of on-farm production efficiency of dairy cows. Due to the lack of information about the variability of regional production efficiency and its determinants, this paper attempts to answer the following questions.

1. Which structural characteristics significantly demarcate fully efficient and inefficient specialised dairy farms from the regional point of view?
2. Which type of partial productivity significantly determines the total technical efficiency?
3. Is there any substitution between labour and capital in specialised dairy farms which would significantly affect the labour productivity?
4. How important are current subsidies and rural development subsidies in the technical efficiency of specialised dairy farms?

## 2. Material and Methods

### 2.1. The Geographic Scope

The FADN database (farm accountancy data network), which annually collects farm economic and structural results in the EU member states, provides structural and economic data in standard results. Complete data for 2010-2011 are available in 108 EU regions.

The farms involved were selected by their economic size and type of farming. Types of farming are defined in terms of the relative importance of the different enterprises on the farm. Relative importance itself is measured quantitatively as a proportion of each enterprise's standard output to the farms' total standard output.

The second sampling criterion was the economic size of farms which is one of the criteria utilised to classify agricultural holdings according to the community typology for agricultural holdings. With regulation (EC) no. 1242/2008, the economic size of an agricultural holding is measured as the total standard output (SO) of the holding expressed in Euro. The exchange rates are published by FADN. The sum of all the SOs per hectare of crop and per head of livestock of each holding is a measure of its overall economic size. According to the official definition in accordance with the regulation 1242/2008, “the standard output (SO) is the average monetary value of the agricultural output at farm-gate price of each agricultural product (crop or livestock) in a given region. The SO is calculated by member states per hectare or per head of livestock, by using basic data for a reference period of five successive years. The SO of the holding is calculated as the sum of the SO of each agricultural product present in the holding multiplied by the relevant number of hectares or heads of livestock of the holding.”

This analysis focuses on specialised milk type of farming (code 45 in FADN grouping). This type of farming contains only farms with share of dairy cows more than 75% out of the ruminants on farm. The FADN regions with available data on specialised dairy farming represent 25 EU member states. This paper analyses the average of 2010-2011 because it is not an extreme case unlike the previous crisis period in the dairy sector: 2008-2009.

The FADN database converts sample into universe (field of survey) using special weighting system. According to the FADN methodology, “weighting system is based on the principle of “free expansion”: a weight calculated for the sample applies to each holding of the sample (extrapolating factor). In order to calculate this individual weight, holdings in the sample and in the field of survey are stratified according to the same three criteria: FADN region, type of farming, and economic size class. The individual weight is equal to the ratio between the numbers of holdings, of the same classification cell (FADN region × type of farming × economic size class), in the population and in the sample.” Consider, for example, very large specialist dairy farms in Brittany. If there are 20 farms belonging to this group in the FADN sample and if there are 1000 in the population, then each individual farm in the sample for that group will have a weight of 1000/20 = 50. To calculate weighting factors, it is necessary to have an accurate and up-to-date field of survey. The FADN field of survey is a subset of the EUROSTAT farm structure survey (FSS).

To ensure that the sample of farms adequately reflects the heterogeneity of farm size, the field of observation was stratified before the sample of farms is selected. If this were not done, there would have been a risk that particular categories of farm (say, large dairy farms in one region or small dairy farms in another region) would not be represented adequately (or at all) by the sample.

Table 1 gives information about state affiliation of the analysed regions. The table contains number of farms in universe, that is, the population of specialised dairy farms. It is important to emphasize that authors use data representing the population not the sample.

### 2.2. The Quantitative Methods

The analysis of economic efficiency of specialised dairy farming respects the view on efficiency in utilization of production factors [26, 27]. To determine the level of the production efficiency of farms, the DEA method is applied. The efficiency analysis, by means of the nonparametric frontier function, was initially developed by Farrell [28]. Unlike the parametric approach, the DEA does not require many model assumptions concerning the form of the production function and the distribution of the probability of random components.

A production unit is efficient when there is no other unit maintaining the same level of output with lower level of inputs, or when there is no other unit achieving a higher level of output with the same level of inputs. Units with the highest efficiency are located on the efficient frontier (at the boundary of efficiency). The purpose of the DEA method is to construct a nonparametric envelopment frontier over the data points such that all observed points lie on or below the production frontier. The technical efficiency (TE) estimates vary between 0 (0%) and 1 (100%). The model assumes variable returns to scale (DEAVRS method, data envelopment analysis with variable returns to scale) and inputs minimization, given the values of outputs. The issue of returns to scale concerns what happens to units' outputs when they change the amount of inputs they are using to produce their outputs. Under the assumption of variable returns to scale, a unit found to be inefficient has its efficiency measured relative to other units in the dataset of a similar scale size only. The results distinguish between increasing, constant (effective), and decreasing returns to scale. The DEA under variable returns to scale is known as the BCC (Banker-Charnes-Cooper) model. The BCC model used in this paper is described in more detail by Cooper et al. [29].

The technical efficiency is the only indicator to separate fully efficient and inefficient regions. However, the calculation of technical efficiency involves the key inputs and key outputs in specialized milk farming.

Six inputs and two outputs per weighted average farm are used for efficiency calculation. The indicators are linked to the FADN standard results codes.

- Outputs in EUR: livestock output (SE206), crop output (SE135).
- Land input (SE025—utilised agricultural area in ha).
- Labour input (SE011—actual working time in hours per year).
- Material costs (SE281—seeds and plants, fertilisers, crop protection, other crop specific costs, feed for grazing livestock, feed for pigs and poultry, and other livestock specific costs in EUR).
- Energy costs (SE345—motor fuels and lubricants, electricity, and heating fuels in EUR).
- Capital costs (SE360 depreciation, SE375 rent paid, SE380 interest paid, SE340 machinery and building current costs, SE390 taxes, and other charges on land and buildings in EUR).
- Contract work (SE350—costs linked to work carried out by contractors and to the hire of machinery in EUR).

Efficiency scores were calculated separately for each region. The technical efficiency (TE) score divides the sample into two groups—efficient regions with TE = 1.0 and inefficient regions with TE < 1.0. The statistical procedure tests the differences of structural and economic indicators between the two groups. The farm net value added (FNVA) per AWU (annual work unit) represents the main income indicator in agriculture. AWU is the unit of measurement of the quantity of human work supplied for each farm. This unit is equivalent to the work of one person, full time, for one year. According to the FADN definition, the FNVA is the remuneration for the fixed factors of production (work, land, and capital), whether they are external or family factors. As a result, holdings can be compared irrespective of their family/nonfamily nature of the factors of production employed. Since it includes costs on external factors, it is a convenient technique to compare the different farm structures within the EU-27. The economic indicators also include modified FNVA per AWU, which is defined as the remuneration for paid and unpaid work only.

Statistical procedures for assessment of differences between efficient and inefficient groups are selected depending on the features of the two groups. The degrees of skew, kurtosis, and omnibus normality are tested. Since the choice of appropriate statistical tests varies by the normality and variance assumptions of the sample, some researchers recommend against using a preliminary test on variances. If the two sample sizes are approximately equal, the equal-variance *t*-test can be used. If the ratio of the two sample sizes (larger sample size over the smaller sample size) is equal to or greater than 1.5, it is possible to use the unequal-variance *t*-test [30]. The results of DEA indicate 45 efficient regions and 63 inefficient regions (ratio = 1.4), so the prerequisite for the equal-variance *t*-test is still fulfilled. However, the Aspin-Welch unequal-variance *t*-test is used because the ratio value is close to the threshold.

The two-sample *t*-test compares the distribution between two groups—inefficient regions (*μ*_1_) and efficient regions (*μ*_2_). The null and alternative hypotheses are *H*_0_: mean *μ*_1_ = mean *μ*_2_, *H*_*A*_: mean *μ*_1_ > mean *μ*_2_ (Diff > 0) or mean *μ*_1_ < mean *μ*_2_ (Diff < 0). So, the one-sided test of hypotheses is applied depending on the subjective assumptions about the efficiency determinants. The statistical analysis is processed automatically by NCSS 9 software.  Table 2 contains basic descriptive statistics of farms.

The sample contains regions with relatively small size farms as well as regions with very large farms with more than 1400 dairy cows per 100 hectares and highly intensive farming on average. The specialised dairy farms often use agricultural area for production of own feeds and bedding. The average share of forage crops in the total utilised agricultural area is 76.0% with a minimum of 20.7%.

Specialised dairy farms have either extensive or intensive stocking intensity. So, this paper also evaluates whether the intensity affects production efficiency.

The question about substitution between capital/contract work can be answered using the LC_sub_ indicator

(1) LCsub=TO/LITO/(CC+CW),

where LC_sub_ is substitution between capital/contract work, TO is total output (total agricultural production), LI denotes labour input (actual working time in hours per year), CC denotes capital costs (depreciation, rent paid, interest paid, machinery and building current costs, taxes, and other charges on land and buildings), and CW means contract work (costs linked to work carried out by contractors and to the hire of machinery). The correlation among input variables is tested by means of the Pearson correlation coefficient at 5% level of significance.

The relationship between the LC_sub_ indicator and labour productivity (FNVA per AWU) is quantified using linear regression analysis. White's LM test checks out the presence of heteroskedasticity when doing the regression analysis.

## 3. Results and Discussion

### 3.1. Structure of EU Specialised Milk Farming

While the coverage of the sector is very high in most EU-15 member states, it is generally lower in other member states. Farms in these member states, particularly large farms in Slovakia and the Czech Republic, diversify their activities a lot, so the proportion of specialised farms is not high. The structure of dairy farms in the Czech Republic and in Slovakia has been influenced by historical consequences. Before 1990, the majority of milk production in Czechoslovakia came from large agricultural companies, either state farms or agricultural cooperatives. After 1990, some specialised dairy farms began to emerge. Currently, only about one-quarter of milk is being produced by specialised dairy farms, which is one of the lowest shares in the EU. The rest of production originates from medium and large mixed crop and livestock agricultural enterprises. In comparison with mixed type of breeding, enterprises focusing on milk breeds of cattle reach higher milk yields, lower costs per litre of milk, and better financial results [31].

The share of the sector covered by specialised farms in the FADN is more than 80% in the EU-15 and around 50% in the EU-2 and EU-10. There are big differences in coverage among member states: only 17% of milk production in Slovakia and 19% in the Czech Republic, but full production in Ireland and Finland. Globally, the FADN sample covers 73% of the dairy cows, and the margin and production costs are valid for 78% of EU-27 milk production.

There are large differences among milk farms across the EU. Farms in the EU-15 are in general much larger and have higher yields than in the EU-10 and EU-2. Milk specialised farms in the EU-15 have 52 dairy cows on average, with a milk yield of 6991 kg/cow, producing 364 t of milk per year, whereas in the EU-10 they have 17 dairy cows, with a yield of 5577 kg/cow, and produce 97 t of milk per year. Farm size is even lower in the EU-2 where farms have 7 dairy cows on average, with a yield of 3877 kg/cow, and produce 27 t of milk per year. These data reflect the diversity of milk farm structures in the EU-27, which are linked to the differences in natural potential and also in the social, economic, and regulatory context. In particular, the different national policies on milk quota management are very likely to have had an impact on the level of restructuring within each member state [32].

### 3.2. Feeding and Welfare

In the traditional milk producing areas in the world, it is common practice to use specialized cattle breeds such as the Holstein, managed in intensive systems and fed high quality forages and large amounts of concentrates. In such systems, the cows invariably are milked without their calves, which are often slaughtered soon after birth or are reared artificially. The levels of production achieved in these specialised herds usually exceed 7000 litres per lactation. Unfortunately, these intensive systems are becoming ever more costly due to their almost complete dependence on concentrate feeds which are frequently imported and competed for by other species.

All dairy cattle should be fed a diet that provides sufficient energy, nutrients, and dietary fibre to meet the metabolic requirements in a way that is consistent with digestion. Specialized milk farms especially are heavily dependent on high quality feedings including high level of energy. When diet is changed, there should be carefully controlled transition feeding in order to prevent poor welfare in the cattle. Feeding systems should allow every individual cow to meet its needs for quantity and quality of feed. Except for high quality feedings provided in sufficient quantity, every dairy cow should be provided with drinking water whatever their diet is. This water should be of sufficient quantity to prevent any dehydration (specialized dairy farms are heavily dependent on water consumption) and should be free from repellent odour and taste, harmful infectious agents, toxic substances, and contaminants that can accumulate in body tissue or be excreted in milk. Both indoors and outdoors, continuous access to water should be provided. Automatically regulated troughs and drinker bowls should be installed in the animal houses and farmyards.

It is also necessary to emphasize the very important role of animal welfare in the case of specialized milk farms. Cows living in those farms have to face much higher level of stress than their colleagues living, for example, in farms focused on meat production. The high level of milk production is accompanied by intensive feeding and milking. It is necessary to provide the high level of attention for milk cows' population. Except for high quality feedings and high level of stabling/housing—it is necessary also to take care of individual animal's health.

There should be systems for monitoring the prevalence of many diseases. Also infection is a very dangerous element which can affect milk production. In this case, the big problem of European countries is lameness. To eliminate the occurrence of lameness, it is necessary to focus the attention on scoring locomotion and foot lesions every 3 to 6 months in all dairy herds. Because of the high risk of lameness in dairy cattle, all dairy farmers should implement a lameness prevention program. On farms with a high prevalence of recognisable locomotor difficulties, for example, approaching 10%, there should be improvement of housing conditions, genetic strain, and management practices.

In addition to improved methods for genetic selection, the prevalence of mastitis should be reduced also through treatment of clinical and subclinical disease, dry cow therapy, identification and elimination of carrier cows, prevention of transmission of infection from cow to cow or through the environment, and improvement of the immune system by minimising stress factors and by a controlled and nutritionally balanced feed intake.

Pain management should be part of the treatment of severe lameness and clinical mastitis.

Farmers should be well trained in recognizing signs of disease at early stages and veterinary advice should be sought at an early stage of disease in dairy cattle. Recommendations on this opinion for disease prevention and management should be followed.

The body of research on dairy cattle welfare should be incorporated into codes of practice and monitoring protocols that address potential hazards and incorporate animal-based measures of welfare outcomes [33].

The international comparison of welfare data is not well available. There is a challenge for survey based on standardized measures of welfare. Broom [34] presents summary of most welfare measures. The best estimate of biological fitness is lifetime reproductive success. Livestock production under bad welfare can suffer from delayed onset of reproduction during development, lengthened intervals between successive breeding, reduced litter size, and early death. Moreover, measures of body damage, such as broken bones or wounds, frequency of indigestions, susceptibility to disease, measurements of grooming, and feeding behavioural responses to difficulties, are good indicators of welfare assessment. Broom also suggests animal life expectancy, responsiveness, and stereotypies as welfare indicators.

Useful system for welfare assessment could also be welfare quality system, funded by EU project FOOD-CT-2004-506508. The system checks four welfare principles. Each principle comprises two to four criteria. Criteria are independent of each other.

- Good feeding: absence of prolonged hunger and absence of prolonged thirst.
- Good housing: comfort around resting, thermal comfort, and ease of movement.
- Good health: absence of injuries, absence of disease, and absence of pain induced by management procedures.
- Appropriate behaviour: expression of social behaviours, expression of other behaviours, good human-animal relationship, and positive emotional state.

### 3.3. Results of the Technical Efficiency

The results in Table 3 confirm the theoretical assumption about returns to scale.

As a business grows, the company initially increases its scale efficiency. After achieving the optimum size, its scale efficiency gradually decreases. Decreasing returns to scale were noted in 2011 in the Czech Republic, Slovakia, Estonia, regions in former East Germany (Brandenburg, Mecklenburg-Vorpommern, Sachsen, Sachsen-Anhalt, and Thueringen), Saarland, two regions in France (Bourgogne and Lorraine), two regions in Italy (Friuli-Venezia and Sicilia), three regions in Hungary (Nyugat-Dunántúl, Észak-Alföld, and Dél-Alföldin), and three regions in the United Kingdom (east of England, west of England, and Scotland). This means that output increases were less than the proportional change in inputs. Nevertheless, not all the regions with decreasing returns to scale have large average farms, for example, regions in France, Italy, and Hungary. Efficient specialised dairy farming with decreasing returns to scale is typical for the Czech Republic, Slovakia, Hungary (Nyugat-Dunántúl), Germany (Brandenburg, Mecklenburg-Vorpommern, Sachsen, Sachsen-Anhalt, and Thueringen), and the United Kingdom (east of England, west of England, and Scotland).

All the regions with efficient returns to scale are fully technically effective (TE = 1.0). The optimum-sized efficient regions are in the “old” EU member states—in France (Picardie, Haute-Normandie, Nord-Pas-de-Calais, and Bretagne), Italy (Lombardia, Liguria, Emilia-Romagna, Umbria, Campania, and Sardegna), the Netherlands, Denmark, the United Kingdom (north of England), and Spain (Castilla-León and Andalucia). The optimal average size of efficient regions in the “new” member states is in Malta and Romania (Nord-Est, Sud-Vest Oltenia, Nord-Vest, and Bucuresti-Ilfov).

Most regions have increasing returns to scale and technically inefficient production. These inefficient regions have 116.0 ESU and 40.8 dairy cows per farm on average. Inefficient regions with increasing returns to scale exist in all geographical parts of Europe.

Table 4 contains economic indicators and the results of a two-sample *t*-test. The economic indicators cover input and output variables including current subsidies.

In efficient regions, the average size of efficient farms (see the Appendix) is significantly higher than in inefficient regions. The utilised agricultural area, economic size, and herd size per farm are higher on average in efficient regions than in inefficient regions. This is consistent with research by Latruffe et al. [13] and Hussien [35]. The dairy farms in efficient regions use more labour input which indicates higher farming intensity.

Regarding the production, the test proves that the efficient regions have a significantly higher crop output per hectare; however, livestock output per livestock unit is not significantly higher in efficient regions. This can be due to the differences in livestock density between efficient and inefficient regions. The inefficient regions have approximately the same share of another output in total output as efficient regions. So, the inefficient regions do not compensate the lower agricultural production by a higher share of nonagricultural activities. Diversification into nonagricultural activities is not a fundamental strategy of inefficient specialised dairy farms.

The more efficient input-output ratio of efficient regions has a positive impact on the significantly favourable share of intermediate consumption to total output. This means that efficient regions spend less specific costs and overhead costs per one unit of output. The differences of total output per total input and per intermediate consumption is significant only at the 10% significance level. Moreover, efficient regions spend slightly more specific livestock costs per hectare (feed, bedding, and veterinary costs) which, on the other hand, generates higher livestock output, though differences in cost intensity are not statistically significant.

The hypotheses about partial factor productivity verify whether the efficient regions have higher productivity of all production factors than inefficient regions. Table 4 shows that efficient regions have significantly higher total output per energy costs, capital costs (at *α* = 0.01), land, and labour (at *α* = 0.05) than inefficient regions. Differences of material and contracting work productivity are significantly higher only at the 10% significance level. The input productivity raises a question of substitution among inputs.  Table 5 provides possible answer.

The correlation matrix in Table 5 indicates the lowest correlation coefficients between contract work and labour and between capital costs and labour. This indicates the presence of capital-labour substitution or contract work-labour substitution among regions. The hypothesis is that substitution of labour for capital or contract work indicates higher technology levels, which can increase labour productivity and farm income.

**Figure 1:**
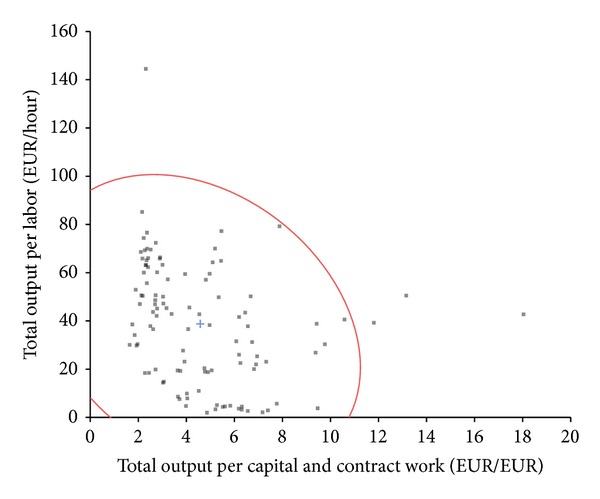
Relationship between labour productivity and capital/contract work productivity.

Figure 1 shows the correlation between capital/contract work productivity and labour productivity. Spearman's rank correlation coefficient between labour productivity and capital/contract work productivity is −0.432 (*P* value = 0.0000). The Pearson correlation coefficient is −0.291 (*P* value = 0.0022). The differences between both correlation coefficients indicate a nonlinear substitution effect.

The higher the LC_sub_ indicator is, the more the labour is substituted for, either by capital or by contract work. Regions in western and northern Europe have the highest LC_sub_ indicators, so they use more capital or contract work. In the 1st/top quartile of LC_sub_ (>18.48), there are regions in Denmark, France, Sweden, the Netherlands, Luxembourg, and Germany. At the other extreme, regions in central, southern, and eastern Europe have the lowest LC_sub_ indicators (<3.44); thus, they use more labour force on the farm. In the 4th/bottom quartile of LC_sub_, there are regions in Italy, Spain, Portugal, Malta, Poland, Latvia, Lithuania, Bulgaria, and Romania.

Table 6 contains the results of a linear regression analysis between FNVA/AWU (in thousands EUR) as dependent variable *y* and indicator LC_sub_ as independent variable *x*.

The adjusted *R* ^2^ shows that the LC_sub_ indicator is not a valuable determinant of farm income level for specialised dairy farming, because it indicates the variability of FNVA per AWU by only 31.8%. It has been mentioned in the literature, however, that the LC_sub_ indicator is a significant determinant of FNVA/AWU in mixed crop and livestock farming with adjusted *R* ^2^ = 0.74 [36] because crop production is not as demanding on own labour as livestock production and substitution of labour by capital or external contract work is more common. Crop production also uses many seasonal workers, whereas livestock production needs full time service for delivering feeds and bedding and for milking. Of course, some dairy farms use robotic milking, but this technology also requires some workers for maintaining the safety and quality of milking process.

**Figure 2:**
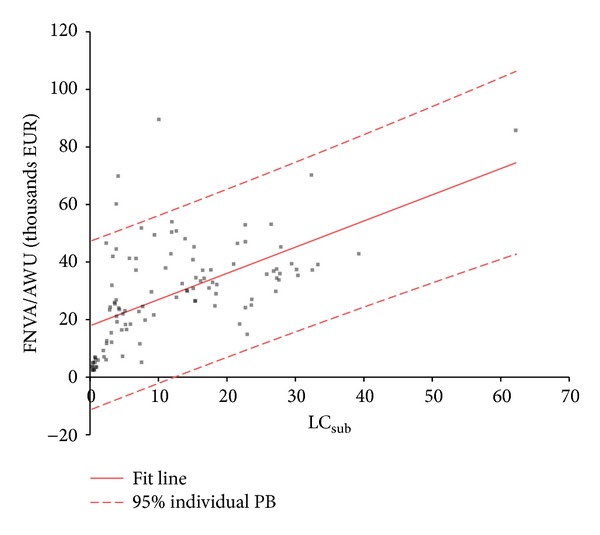
Regression line.

Figure 2 visually presents the regression function.

Table 7 presents the differences in FNVA/AWU and subsidies.

The FNVA per AWU differs between efficient and inefficient regions. Efficient regions are characterised by significantly higher income per AWU and per hectare. Inefficient regions, however, receive significantly higher current subsidies per total output because they produce less total output per average farm. Total current subsidies per hectare do not significantly differ. An important finding is that inefficient regions receive more rural development subsidies than efficient regions. The production function includes only commodity outputs, and production of the noncommodity outputs (public goods) actually leads to a decrease in technical efficiency, since agricultural enterprises have higher costs and/or achieve lower production [5]. Rural development subsidies include payments in compensation for farming in less favoured areas as well as environmental subsidies. Higher rural development subsidies per total output and hectare in inefficient regions point to much more important production of public goods, such as maintaining landscape or environmentally friendly production [37]. Differences in rural development subsidies indicate that inefficient regions farm more extensively and produce more public goods.

Table 8 depicts the structural characteristics of efficient and inefficient dairy farms in EU regions. The table focuses on differences in structure of crop and livestock production, labour productivity, livestock intensity, use of hired external factors, and indebtedness.

Efficient regions have significantly higher labour input per hectare but not per dairy cow. It is caused by significantly higher livestock intensity as seen from number of livestock units per 100 hectares. Average stocking intensity in inefficient regions is 1.7 livestock units per hectare of forage crops, whereas on average it is 2.5 livestock units in efficient regions. It also confirms more intensive production in efficient regions.

The efficiency of specialised dairy farms does not significantly depend on crop structure. Only the share of set-aside land in total agricultural area is significantly higher in inefficient regions. Moreover, there are no significant differences in livestock structure. The milk yield is slightly higher in efficient regions but the differences are not statistically significant.

The share of hired labour does significantly differ between efficient and inefficient regions. Moreover, efficient regions have higher total production per contract worker; thus, the hired labour is more effectively used in efficient regions. A larger share of hired labour in efficient regions is related to the larger size of dairy farms in the efficient group of regions. Simultaneously, a larger share of hired labour implies a more industrialised form of milk production in larger efficient farms. The use of external capital and rented utilised agricultural area does not significantly differ between efficient and inefficient regions in the EU.

## 4. Conclusions

The aim of the paper was to assess the production efficiency of specialised dairy farms among the FADN EU regions in 2010-2011 and to determine the structural and economic determinants of production efficiency. The analysis of 108 EU regions with available data on specialised dairy farms was processed by the DEA method and the Aspin-Welch *t*-test of statistical hypotheses. The paper revealed some significant determinants of regional production efficiency and income level.

- The analysis of technical efficiency of specialised dairy farms revealed 45 efficient regions and 63 inefficient regions. So, the variability of regional technical efficiency is much lower than in the case of individual-farm analysis. There are generally larger farms in efficient regions on average. In central Europe, specialised dairy farms in the Czech Republic, Slovakia, and one region in Hungary are technically efficient. All four regions in Poland are technically inefficient with increasing returns to scale.
- The theoretical assumptions about scale efficiency have been verified. All regions with optimal returns to scale are efficient. Decreasing returns to scale are typical for regions with the largest farms on average, such as the Czech Republic, Slovakia, and regions in the former East Germany.
- The analysis of partial productivity shows that land and labour productivity demarcate the efficient and inefficient regions at the 5% significance level. The productivity of energy inputs and capital costs are key determinants of specialised dairy farm efficiency at 99% significance level. Moreover, efficient regions have a significantly higher FNVA per AWU and hectare, which is in compliance with the assumptions. Alternatively, the milk yield is slightly higher in efficient regions but the differences are not statistically significant.
- Subsidies on rural development are significantly higher per total output as well as per hectare in inefficient regions. The inefficient regions provide more public goods for rural development, which are generally produced with higher costs and/or lower production. Moreover, the structural indicators show that higher farming intensity significantly increases the production efficiency. This corresponds to recent research carried out by other authors at the farm level.
- The results prove moderate substitution between labour and capital/contract work. The proposed indicator LC_sub_, as the share of labour productivity to capital/contract work productivity, significantly determines the FNVA per AWU in specialised dairy farms. Nevertheless, the LC_sub_ indicator explains a variability of FNVA per AWU by only 31.8%, which is much lower than in the case of mixed crop and livestock farms. It can be concluded that specialised dairy farms do not substitute labour by contractual work or new technology in such dimensions as mixed crop and livestock farms, because livestock production requires a certain level of full-time control staff to ensure safety and welfare of dairy cows.

## Figures and Tables

**Table 1:** Regions (108) and number of farms represented (population of specialised dairy farming).

Undifferentiated member states (FADN regions)	Austria (27040), Czech Republic (980), Denmark (3880), Estonia (1460), Ireland (15600), Lithuania (18100), Luxembourg (580), Latvia (8210), Malta (110), The Netherlands (17400), Slovakia (380), and Slovenia (6330)

FADN regions within member states	

Belgium	Vlaanderen (3290), Wallonie (2060)

Bulgaria	Severozapaden (2710), Severen tsentralen (2150), Severoiztochen (1290), Yugozapaden (2470), Yuzhen tsentralen (6190), and Yugoiztochen (2150)

Finland	Etela-Suomi (2410), Sisa-Suomi (3340), Pohjanmaa (2010), and Pohjois-Suomi (2240)

France	Champagne-Ardenne (690), Picardie (1050), Haute-Normandie (1200), Centre (600), Basse-Normandie (5450), Bourgogne (380), Nord-Pas-de-Calais (1940), Lorraine (1750), Alsace (520), Franche-Comté (3560), Pays de la Loire (5900), Bretagne (10140), Poitou-Charentes (910), Aquitaine (1510), Midi-Pyrénées (2290), Rhônes-Alpes (4990), Auvergne (4090), and Languedoc-Roussillon (360)

Germany	Schleswig-Holstein (3600), Niedersachsen (8410), Nordrhein-Westfalen (4910), Hessen (2520), Rheinland-Pfalz (1820), Baden-Württemberg (6890), Bayern (32270), Saarland (200), Brandenburg (370), Mecklenburg-Vorpommern (410), Sachsen (740), Sachsen-Anhalt (310), and Thueringen (240)

Hungary	Nyugat-Dunántúl (330), Észak-Alföld (1150), and Dél-Alföld (980)

Italy	Aosta (660), Piemonte (1860), Lombardia (5280), Trentino (670), Alto-Adige (5440), Veneto (3020), Friuli-Venezia (860), Liguria (140), Emilia-Romagna (3420), Umbria (180), Lazio (1700), Abruzzo (620), Molise (820), Campania (2770), Puglia (1900), Basilicata (390), Sicilia (1330), and Sardegna (720)

Poland	Pomorze and Mazury (10870), Wielkopolska and Slask (14400), Mazowsze and Podlasie (66900), and Malopolska and Pogórze (11690)

Portugal	Norte e Centro (4750), Açores (2800)

Romania	Nord-Est (29540), Sud-Est (21510), Sud-Muntenia (7240), Sud-Vest Oltenia (19410), Vest (6850), Nord-Vest (27250), Centru (15150), and Bucuresti-Ilfov (570)

Spain	Galicia (11660), Asturias (2330), Cantabria (1650), Pais Vasco (430), Navarra (220), Cataluna (730), Baleares (190), Castilla-León (1710), and Andalucia (680)

Sweden	Slattbygdslan (2780), Skogs-och mellanbygdslan (1730), and Lan i norra (900)

United Kingdom	North of England (2430), east of England (1080), west of England (4000), Wales (2000), Scotland (1120), and Northern Ireland (3350)

Source: authors based on the FADN database.

**Table 2:** Basic descriptive statistics of farms in 2011 (*N* = 108).

Variable	Mean	Standard deviation	Minimum	Maximum
Crop output (EUR)	40 390.88	66 481.40	995.00	375 711.00
Livestock output (EUR)	172 397.74	178 183.58	4 348.00	933 218.00
Utilised agricultural area (ha)	89.91	140.85	1.52	967.02
Milk yield (kg/cow/year)	6 440.03	1 774.72	2 480.64	9 352.46
Labour input (AWU)	2.78	3.28	1.06	26.75
Economics size (ESU∗)	181.91	215.95	6.00	1 158.40
Livestock units (LU∗∗) per 100 ha	179.78	246.86	38.27	2 494.85
Dairy cows units per 100 ha	113.93	151.80	21.35	1 489.18
Stocking intensity (LU)/ha of forage crops)	2.04	1.98	0.48	19.87

Notes: *ESU (economic size unit) = 1 ESU is 1000 EUR of standard output. **LU (livestock unit)-converting average number of animals to livestock units is done applying to this number a coefficient related to the category of animal. For example, one dairy cow is one LU.

**Table 3:** Distribution of the returns to scale.

Indicator	Inefficient regions	Efficient regions	Total	Average dairy cows (LU∗)	Average ESU∗∗
Number of regions with decreasing returns to scale	8	11	19	126.86	449.36
Number of regions with efficient returns to scale	0	20	20	55.52	165.94
Number of regions with increasing returns to scale	55	14	69	40.57	112.89

Total	63	45	108	58.52	181.91

Notes: *LU (livestock unit)-converting average number of animals to livestock units is done applying to this number a coefficient related to the category of animal. For example, 1 dairy cow is one LU. **ESU (economic size unit) = 1 ESU is 1000 EUR of standard output.

**Table 4:** Differences in economic indicators.

Indicator	Unit	Inefficient regions (*μ*_1_), *N* = 63	Efficient regions (*μ*_2_), *N* = 45	*H*_0_ (*μ*_1_ − *μ*_2_)	*t* -statistic	*P* value	Sig.
Utilised agricultural area	ha/farm	58.53	133.83	Diff < 0	−2.418	0.0098	∗∗∗
	SD∗	41.69	205.95				

Economic size	ESU/farm	119.92	268.69	Diff < 0	−3.215	0.0012	∗∗∗
	SD	74.17	304.05				

Labour input (hours per year)	hours/farm	4 271.34	8 065.80	Diff < 0	−2.605	0.0062	∗∗∗
	SD	1 030.79	9 733.53				

Dairy cows	LU/farm	41.77	81.96	Diff < 0	−3.469	0.0005	∗∗∗
	SD	22.97	75.24				

Crop output	EUR/ha	442.40	659.98	Diff < 0	−2.626	0.0053	∗∗∗
	SD	314.32	488.13				

Livestock output	EUR/LU	1 607.62	1 697.18	Diff < 0	−0.989	0.1628	—
	SD	440.04	480.65				

Other production in Total input	%	3.297	3.217	Diff > 0	0.090	0.4642	—
	SD	4.472	4.635				

Total output per total input	EUR/EUR	1.167	1.290	Diff < 0	−1.616	0.0557	∗
	SD	0.234	0.468				

Total output per total intermediate consumption	EUR/EUR	1.583	1.756	Diff < 0	−1.644	0.0528	∗
	SD	0.316	0.655				

Total output per hectare	EUR/ha	2 787.04	4 970.83	Diff < 0	−1.789	0.0401	∗∗
	SD	1 570.33	8 079.22				

Total output per working hour	EUR/hour	34.34	44.99	Diff < 0	−2.111	0.0190	∗∗
	SD	20.81	28.90				

Total output per material costs	EUR/EUR	2.463	2.698	Diff < 0	−1.413	0.0813	∗
	SD	0.585	1.003				

Total output per energy costs	EUR/EUR	14.51	17.70	Diff < 0	−3.441	0.0005	∗∗∗
	SD	3.87	5.28				

Total output per capital costs	EUR/EUR	4.623	6.221	Diff < 0	−2.580	0.0062	∗∗∗
	SD	1.877	3.841				

Total output per contracting work	EUR/EUR	60.39	270.95	Diff < 0	−1.305	0.0993	∗
	SD	83.37	1 080.12				

Specific livestock costs per LU∗∗	EUR/LU	715.70	746.93	Diff < 0	−0.477	0.3174	—
	SD	293.64	362.76				

Notes: *SD = standard deviation; **LU (livestock unit)-converting average number of animals to livestock units is done applying to this number a coefficient related to the category of animal. For example, 1 dairy cow is one LU.

∗∗∗Means significance at *α* = 0.01.

**Table 5:** Pearson correlation among input variables (*N* = 108).

	Land	Labour	Material	Energy	Capital	Contract
Land	1.000	0.936	0.864	0.943	0.885	0.861
Labour	0.936	1.000	0.832	0.936	**0.804**	**0.720**
Material	0.864	0.832	1.000	0.935	0.931	0.858
Energy	0.943	0.936	0.935	1.000	0.935	0.825
Capital	0.885	**0.804**	0.931	0.935	1.000	0.920
Contract	0.861	**0.720**	0.858	0.825	0.920	1.000

Note: all correlation coefficients are statistically significant at *α* = 0.01.

**Table 6:** Regression between income indicators FNVA/AWU (thousand EUR) and LC_sub_.

Regression	Adj. *R* ^2^	*P* value	Standard error	White test LM (*P* value)
*y* = 17.87289 + 0.90993*x*	0.31779	<0.0001	14.62732	1.24513 (0.53657)

Notes: variable “*y*” denotes farm net value added per annual work unit (FNVA/AWU); “*x*” denotes LC_sub_ variable specified in (1). There is no heteroskedasticity in linear regression model.

**Table 7:** Differences in income indicator FNVA/AWU and subsidies.

Indicator	Unit	Inefficient regions (*μ*_1_), *N* = 63	Efficient regions (*μ*_2_), *N* = 45	*H*_0_ (*μ*_1_ − *μ*_2_)	*t*-statistic	*P* value	Sig.
Total current subsidies per total output	EUR/EUR	0.1930	0.1532	Diff > 0	2.157	0.0167	∗∗
	SD∗	0.1076	0.0839				

Total current subsidies per hectare	EUR/ha	471.49	554.44	Diff < 0	−0.712	0.2398	—
	SD	249.23	752.26				

Rural development subsidies∗∗ per total output	EUR/EUR	0.0484	0.0293	Diff > 0	1.909	0.0295	∗∗
	SD	0.0637	0.0405				

Rural development subsidies per hectare	EUR/ha	109.89	70.25	Diff > 0	1.870	0.0322	∗∗
	SD	140.57	78.10				

Investment subsidies per hectare	EUR/ha	32.11	84.31	Diff < 0	−0.812	0.2105	—
	SD	42.82	429.64				

Farm net value added (FNVA) per AWU∗∗∗	EUR/AWU	26 191.02	33 146.04	Diff < 0	−1.916	0.0297	∗∗
	SD	14 242.59	21 165.67				

Farm net value added (FNVA) per hectare	EUR/ha	1 070.43	1 838.08	Diff < 0	−2.699	0.0047	∗∗∗
	SD	621.95	1 834.50				

Farm net value added (FNVA) per livestock unit	EUR/LU	784.86	887.44	Diff < 0	−1.397	0.0835	∗
	SD	266.74	437.77				

Note: *SD = standard deviation. **Rural development subsidies = environmental subsidies + payments on less favoured areas + other rural development subsidies. ***AWU = annual work unit.

**Table 8:** Structural determinants of production efficiency in EU regions.

Indicator	Unit	Inefficient regions (*μ*_1_), *N* = 63	Efficient regions (*μ*_2_), *N* = 45	*H*_0_ (*μ*_1_ − *μ*_2_)	*t*-statistic	*P* value	Sig.
Labour input per hectare	hours/ha	130.66	268.71	Diff < 0	−1.897	0.0320	∗∗
	SD∗	114.99	478.42				

Labour input per dairy cow	hours/LU∗∗	155.73	182.32	Diff < 0	−0.691	0.2460	—
	SD	126.67	234.76				

Cereals in UAA∗∗∗	%	18.43	20.16	Diff < 0	−0.649	0.2591	—
	SD	10.37	15.58				

Other field crops in UAA	%	2.16	3.15	Diff < 0	−1.462	0.0748	∗
	SD	1.81	4.27				

Forage crops in UAA	%	76.57	75.23	Diff > 0	0.412	0.3407	—
	SD	13.70	18.62				

Set-aside land per total agricultural area	%	0.497	0.262	Diff > 0	1.693	0.0467	∗∗
	SD	0.899	0.537				

Dairy cows per total LU	%	62.27	62.92	Diff < 0	−0.484	0.3149	—
	SD	6.94	6.88				

Other cattle per Total LU	%	36.56	35.70	Diff > 0	0.603	0.2741	—
	SD	6.99	7.52				

Pigs per total LU	%	0.81	1.04	Diff < 0	−0.756	0.2258	—
	SD	1.73	1.48				

Poultry per total LU	%	0.177	0.248	Diff < 0	−0.761	0.2243	—
	SD	0.465	0.490				

Number of LU per 100 hectares	LU/100 ha	141.28	233.69	Diff < 0	−1.660	0.0518	∗
	SD	73.00	368.23				

Number of dairy cows per 100 hectares	LU/100 ha	88.773	149.139	Diff < 0	−1.773	0.0414	∗∗
	SD	49.309	224.585				

Stocking intensity	LU/ha f.c.∗∗∗	1.727	2.489	Diff < 0	−1.731	0.0449	∗∗
	SD	0.793	2.875				

Milk yield per year	kg/cow	6 363.91	6 546.59	Diff < 0	−0.514	0.3044	—
	SD	1 675.36	1 919.47				

Debt ratio	%	17.35	19.91	Diff < 0	−0.734	0.2326	—
	SD	15.36	19.46				

Share of hired labour	%	16.71	29.45	Diff < 0	−2.616	0.0056	∗∗∗
	SD	15.14	30.07				

Share of rented UAA	%	60.45	60.73	Diff < 0	−0.056	0.4779	—
	SD	22.66	27.84				

Notes: *SD = standard deviation; **LU = livestock unit; ***UAA = utilized agricultural area, ***f.c. = forage crops.

## Appendix

### List of Fully Efficient Regions (TE = 1)

Germany: Brandenburg, Mecklenburg-Vorpommern, Sachsen, Sachsen-Anhalt, and Thueringen
France: Picardie, Haute-Normandie, Basse-Normandie, Nord-Pas-de-Calais, Alsace, Franche-Comté, Bretagne, Midi-Pyrénées, Auvergne, and Languedoc-Roussillon
Italy: Lombardia, Alto-Adige, Liguria, Emilia-Romagna, Umbria, Campania, and Sardegna
The Netherlands: Country
Denmark: Country
United Kingdom: North of England, east of England, west of England, and Scotland
Spain: Galicia, Navarra, Cataluna, Castilla-León, and Andalucia
Portugal: Açores
Czech Republic: country
Hungary: Nyugat-Dunántúl
Malta: Country
Slovakia: Country
Bulgaria: Yuzhen Tsentralen
Romania: Nord-Est, Sud-Est, Sud-Vest Oltenia, Vest, Nord-Vest, and Bucuresti-Ilfov.
